# Recovery and Concentration of Polyphenols from Roasted Hazelnut Skin Extract Using Macroporous Resins

**DOI:** 10.3390/foods11131969

**Published:** 2022-07-02

**Authors:** Negin Seif Zadeh, Giuseppe Zeppa

**Affiliations:** Department of Agriculture, Forest and Food Sciences (DISAFA), University of Turin, 10095 Grugliasco, Italy; negin.seifzadeh@unito.it

**Keywords:** Amberlite resin, hazelnut skin, polyphenols, by-product

## Abstract

Hazelnut skin is a rich source of polyphenols but is generally discarded during the roasting process of hazelnuts. Previous studies reported the extraction and identification of these compounds using different solvents and procedures; however, there are few reports on their enrichment and purification. In this study, three types of Amberlite macroporous resins (XAD 16, XAD 4, and XAD 7) were compared to evaluate the enrichment of polyphenols via adsorption and desorption mechanisms. The operating condition parameters for polyphenol adsorption/desorption of each resin were determined, the kinetics of adsorption were examined, and a method for polyphenol recovery was developed using static and dynamic adsorption/desorption. Antioxidant activity and high-performance liquid chromatography-diode array detection were used to confirm the increase in polyphenols obtained using the adsorption/desorption technique. XAD16 showed the highest adsorption capacity, with a recovery of 87.7%, and the adsorption kinetics fit well with a pseudo-second-order model. The highest poly-phenol desorption ratio was observed using an ethanol/water solution (70% *v*/*v*) at a flow rate of 1.5 bed volume/h.

## 1. Introduction

Hazelnut is one of the most widely consumed nut crops. Its worldwide production in 2020 was reported as 528,070 tons, with Turkey, Italy, and Spain as the major producers [1]. Approximately 50–60% of the nut is discarded as by-products, such as shell, skin, and damaged nuts, during the dehulling, roasting, and sorting processes [2]. Many parts of these by-products are rich in bioactive compounds, particularly polyphenols, which can be extracted and used in the food, feed, cosmetic, and pharmaceutical industries [3]. The skin is considered as one of the most useful components of hazelnut by-products, accounting for 2.5% of the total weight of the nut which is separated from the kernel during the roasting process [4]. The antioxidant capacity of hazelnut skin is significantly higher than that of the hazelnut [5], and the roasted skin is even richer in total phenols and has higher antioxidant activity compared to in the natural skin [6,7,8]. Recovering bioactive compounds from hazelnut skin would increase the availability of large amounts of molecules of natural origin and positively impact disposal management, providing considerable economic advantages, such as minimizing the challenges of waste management occurring due to the lack of proper disposal sites to avoid the spread of insects and unwanted wildlife, and production of value-added products from low-cost material. Polyphenols can be extracted from plants using solvents or supercritical fluids. Although the use of solvents is less costly and simpler, it is not selective and results in diluted extracts with low polyphenol concentrations. Macroporous resins are physiochemically stable polymers with polar, non-polar, or slightly polar characteristics and high adsorption capacities for organic compounds [9]. They can be used to purify and concentrate active compounds from complex extracts [10]. The target molecules in aqueous and non-aqueous solutions can be adsorbed by macroporous resins via electrostatic forces, hydrogen bonding interactions, complexation, and size sieving [3]. Resins with large surface areas and pore sizes provide numerous active sites that can interact with the target molecules, and thus, are useful for extracting active compounds. The extraction and regeneration processes are simple and inexpensive [11]. In this study, three poly aromatic Amberlite resins (XAD 4, XAD 7, and XAD 16) were used to concentrate and purify polyphenols in the ethanol extracts of roasted hazelnut skins. Amberlite XAD 16 and XAD 4 is hydrophobic, whereas Amberlite XAD 7 is moderately hydrophilic. Strongly polar resins contain sulfur or nitrogen oxide groups and are not suitable for purifying polyphenols. Slightly polar macroporous resins such as XAD 7 are generally composed of polyacrylate polymers with multifunctional methacrylate crosslinking agents, whereas non-polar macroporous resins such as XAD 16 and XAD 4 consist of styrene and divinylbenzene polymers and are suitable for separating weakly polar compounds [10]. The objective of this research is to propose an efficient method of recovering and purifying polyphenols from hazelnut skin extract, comparing three Amberlite resins and optimizing the operating condition parameters of polyphenol adsorption/desorption. For this purpose, the static experiments were performed to select the best resin and solvent and the kinetics of adsorption were studied. The optimum flow rates were selected through dynamic adsorption/desorption and validation of method for polyphenols recovery from hazelnut skin extract has been measured comparing the phenolic content and antioxidant activity of the crude and concentrated extracts.

## 2. Materials and Methods

### 2.1. Materials

Roasted hazelnut skin of Tonda Gentile delle Langhe PGI was provided by La Gentile srl (Cortemilia, Italy) and obtained by roasting for 7 min at 190 °C. All chemicals used were of analytical grade. Folin–Ciocalteu phenol reagent (2 M), 2,2-diphenyl-1-picrylhydrazyl (DPPH), 6-hydroxy-2,5,7,8-tetramethylchroman-2-carboxylic acid (97%; Trolox), methanol (99.9%), formic acid (98–100%), and ethanol (99.9%) were provided by Sigma-Aldrich (St. Louis, MO, USA). High-performance liquid chromatography (HPLC)-grade standards (gallic acid, protocatechuic acid, catechin, epicatechin, and quercetin) were purchased from Fluka (Buchs, Switzerland). Ultrapure water was prepared using a Milli-Q filter system (Millipore, Billerica, MA, USA). Amberlite macroporous resins (XAD 4, XAD 7, and XAD 16) were supplied by Sigma-Aldrich (Table 1). Before use, the resins (50 g) were soaked in pure ethanol (100 mL) for 3 h at 22 °C with constant rotatory agitation on a VDRL 711 orbital shaker (Asal S.r.l, Milan, Italy) at 60 rpm. The ethanol was removed, and the resins were rinsed with excess ultra-pure water.

### 2.2. Polyphenol Extraction

Polyphenols were extracted from hazelnut skin as described by Locatelli et al. [6]. Prior to extraction, the hazelnut skin was defatted with *n*-hexane using the method described by Özdemir et al. [12]. Defatted hazelnut skins (2 g) were extracted with 50 mL of pure ethanol at 22 °C for 1 h in the dark using a VDRL 711 orbital shaker under constant rotatory agitation at 60 rpm. The extract was centrifugated at 2800× *g* for 10 min at 4 °C. The solid residue was re-extracted for 30 min with 25 mL of pure ethanol and then centrifuged. The two supernatants were mixed, filtered through a 0.45 μm nylon membrane, and stored in amber vials at −18 °C until analysis. All extractions were performed in triplicate.

### 2.3. Total Polyphenolic Content

The total phenolic content (TPC) of the extracts was determined spectrophotometrically as described by Barbosa-Pereira et al. [13] using a BioTek Synergy HT spectrophotometric multi-detection 96-well microplate reader (BioTek Instruments, Winooski, VT, USA). The absorbance was measured in triplicate at 740 nm. A standard curve of gallic acid (100–600 μM; R^2^ = 0.9994) was used to quantify the phenolic content, which was expressed as milligrams of gallic acid equivalents per milliliter of the fresh extract (mg GAE/mL).

### 2.4. Antioxidant Capacity

The antioxidant capacity of the extracts was determined using the DPPH^•^ radical scavenging method with a BioTek Synergy HT spectrophotometric multidetection 96-well microplate reader [13]. The decrease in DPPH absorbance was measured at 515 nm. The antioxidant capacity was expressed as the inhibition percentage (IP) of DPPH radicals and was calculated using the following equation:

% Inhibition = ((A_0_ − A_e_)/A_0_) × 100 (1)

where A_0_ is the absorbance of the blank and A_e_ is the absorbance at 30 min. A standard curve of Trolox (12.5–350 μM; R^2^ = 0.9982) was used to determine the radical-scavenging activity, and the results are expressed as micromoles of Trolox equivalent per mL of extract. Three analyses were evaluated for each sample.

### 2.5. ABTS^+^ Assay

The antioxidant capacity of the extracts was determined using the ABTS^•+^ assay as described by Re et al. [14] with some modifications. An aliquot of ABTS solution (7 mM) was reacted with 2.45 mM potassium persulfate to produce an ABTS radical cation (ABTS^+^). The solution was incubated in the dark for 12 h at 22 °C to ensure its stability. Before use, the ABTS^+^ stock solution was diluted with ethanol to an absorbance of 0.70 ± 0.02 at 734 nm and 30 °C. The diluted ABTS^+^ solution (3 mL) was mixed with 30 μL of sample, and the absorbance was measured after 6 min. The ABTS^+^ scavenging activity was calculated using the following equation:

% Inhibition = ((A_0_ − A_e_)/A_0_) × 100 (2)

where A_0_ is the absorbance of the blank and A_e_ is the absorbance at 6 min. The results were expressed as μM Trolox equivalent (TE)/mL of extract, using a dose–response curve for Trolox (0–350 μM; R^2^ = 0.996) as the standard. Each sample was evaluated in triplicate.

### 2.6. Reversed Phase-HPLC-Diode Array Detector Analysis

A reversed-phase HPLC coupled with a Thermo-Finnigan Spectra System diode array detector (Thermo-Finnigan, Waltham, MA, USA) was used to quantify the phenolic components in the extracts. The instrument was equipped with an SCM 1000 degasser, an AS 3000 automatic injector, a P2000 binary gradient pump, and a Finnigan Surveyor PDA Plus detector. ChromQuest software (version 5.0; Thermo-Finnigan, Waltham, MA, USA) was used for data acquisition. Separation was performed using a reverse-phase Kinetex Phenyl-Hexyl C18 column (150 × 4.6 mm id and 5 μm particle size; Phenomenex, Torrance, CA, USA) at a flow rate of 1 mL/min. The mobile phase was composed of formic acid at 0.1% *v*/*v* (A) and acetonitrile (B), and the sample injection volume was 10 μL. The following gradient elution was utilized: 5% B for 0–7 min; a linear gradient from 5% to 50% B for 7–35 min; a linear gradient from 50% to 80% B for 35–37 min; a linear gradient from 80% to 90% B for 37–38 min; and a linear gradient until 90% A and 10% B were reached for 38–41 min. Gallic acid, protocatechuic acid, catechin and epicatechin, and quercetin were quantified by measuring the absorbance at 271, 293, 279, and 366 nm, respectively. Quantification was performed using the external standard linear calibration curves obtained under the same conditions.

### 2.7. Static Adsorption/Desorption Evaluation

To define the adsorption capacity of the resins for polyphenolic compounds, five amounts of activated macroporous resin (1, 2, 3, 4, and 5 g) were placed in 100 mL flasks and 50 mL of hazelnut skin extract was added. The sealed flasks were shaken at 22 °C for 24 h in the dark using a VDRL 711 orbital shaker under constant rotatory agitation at 120 rpm. TPC was evaluated before and after adsorption, and the adsorption capacity and adsorption ratio were calculated using the following equations:

Adsorption capacity:

q_a_ = ((C_0_ − C_e_) × V_i_)/M (3)

Adsorption ratio:

A (%) = ((C_0_ − C_e_)/C_0_) × 100 (4)

where q_a_ is the adsorption capacity (mg/g dry resin), C_0_ is the TPC value of the extract before the adsorption phase (mg GAE/mL), C_e_ is the TPC value of the extract after the adsorption phase (mg GAE/mL), V_i_ is the volume of the extract (mL), and M is the weight of the resin (g).

After adsorption, the resins were placed in a 100 mL flask and treated with 50 mL of three ethanol solutions (50%, 70%, and 99.9% *v*/*v*). The sealed flasks were shaken at 22 °C for 24 h in the dark using a VDRL 711 orbital shaker under constant rotatory agitation at 120 rpm. The desorption ratio (D%) was calculated using the following equation:

D (%) = (C_d_ × V_d_)/((C_0_ − C_e_) × V_i_) × 100 (5)

where C_d_ is the TPC value of the ethanol solution after desorption (mg GAE/mL), V_d_ is the volume of the ethanol solution used for the desorption phase (mL), V_i_ is the volume of the extract (mL), C_0_ is the TPC value of the extract before the adsorption phase (mg GAE/mL), and C_e_ is the TPC value of the extract after the adsorption phase (mg GAE/mL).

To evaluate the adsorption kinetics, 1 g of each resin was mixed in a flask containing 50 mL of extract with a TPC value of 5 mg GAE/mL and shaken at 22 °C in the dark on a VDRL 711 orbital shaker under constant rotatory agitation at 120 rpm. The TPC of the solution was analyzed every 15 min for the first 2 h and then every 30 min for 6 h. To evaluate the adsorption kinetics, the obtained results were fitted using two widely used kinetic models, the pseudo-first-order [6] and pseudo-second-order models [7]:

Ln (q_e_ − q_t_) = −k_1_ t + ln q_e_ (6)

t/q_t_ = 1/(k_2_ q^2^_e_) + t/q_e_ (7)

where q_e_ (mg/g) and q_t_ (mg/g) are the adsorption capacities at equilibrium and at time t (min), respectively, and k_1_ (min^−1^) and k_2_ (g/mg min) are the rate constants of the pseudo-first and pseudo-second order models, respectively [15].

The fit of each model to the experimental data was estimated using the linear regression correlation coefficient (R^2^).

### 2.8. Dynamic Adsorption/Desorption

Dynamic adsorption was performed by loading the hazelnut skin extracts (5 mg GAE/mL) into a stainless-steel column (300 × 78 mm) packed with the amount of activated macroporous resin that showed the best adsorption/desorption values in the static experiments; the column was connected to a P2000 Thermo-Finnigan pump to obtain different flow rates (1.5, 3, and 5 bed volumes (BV)/h). Dynamic desorption was performed after dynamic adsorption, and the column was eluted with three different ethanol solutions (50%, 70%, and 99.9% *v*/*v*) at varying flow rates (1.5, 3, 5 BV/h) to recover the adsorbate. The inlet volume for both adsorption and desorption was 10 BV.

### 2.9. Statistical Analysis

The results are expressed as the mean ± standard deviation. Significant differences between means were identified using one-way analysis of variance (ANOVA) followed by Duncan’s post-hoc or two-tailed Student *t*-test. Statistical significance was set at *p* < 0.05. The effect of factor interaction on static desorption was examined using the generalized linear model. The data were analyzed using SPSS version 28.0 software (SPSS Inc., Chicago, IL, USA).

## 3. Results

### 3.1. Static Adsorption/Desorption

The adsorption capacity and desorption ratio are typically regarded as the two main benchmarks for selecting resins. We first selected the best resin type and amount for adsorbing polyphenolic compounds from hazelnut skin extracts. The adsorption ratios and capacities of the resins according to their amounts are shown in Table 2 and Table 3, respectively. The absorption ratio increased with increasing amounts of resin, and the maximum value was obtained using 5 g of resin. XAD 16 and XAD 4 showed better adsorption ratios and capacities compared to that of XAD 7 for all amounts of resin evaluated. Using 5 g of XAD 16, 65.06 ± 0.14% of polyphenols in the extract were adsorbed, and this resin showed the highest adsorption capacity (40.05 ± 0.55 mg GAE/g dry resin). The adsorption capabilities of resins are related to both the target compound and absorbent properties, such as polarity, particle size, surface area, pore diameter, and chemical structure. Particularly, polyphenol compounds can be absorbed by macroporous resins via physical mechanisms, such as van der Waals forces, hydrogen bonds, and π-π conjugation between phenolics and the benzene rings of resins [16]. Polyphenols contain hydrogen groups and benzene rings and, depending on their structure, exhibit different polarities. Although XAD 16 and XAD 4 have similar polarities, XAD 16 provides a higher surface area and pore volume size and absorbs more polyphenols.

The desorption ratios of absorbed polyphenols from resin using different concentrations of ethanol (50%, 70%, and 99.9% *v/v*) are shown in Table 4. A significant two-way interaction was observed (*p* < 0.001), confirming that the change in the number of resins or concentration of solvent affected the amount of polyphenol desorption for each resin type. Because a significant difference was found between each of the two variables and the main effects were significant, one-way ANOVA was performed to compare the differences within each group. During the static adsorption stage, XAD 4 and XAD 16 exhibited the maximum desorption ratio when 70% *v/v* ethanol was used, whereas XAD 7 showed the highest desorption when 50% *v/v* of ethanol was used as the solvent. Non-polar resins showed better adsorption and desorption of polyphenols compared to the slightly polar resin. Using a 70% *v/v* ethanol solution, 76.64% and 81.17% of the polyphenols were desorbed that had been absorbed by 5 g of XAD 4 and XAD 16, respectively, whereas the lowest efficiency was observed when 99.9% ethanol was used to recover the polyphenols from 1 g of XAD 7. Similarly, Wang et al. [10] compared different concentrations of ethanol solution (10–100%) to recover polyphenols adsorbed by HPD-300 (non-polar) resin. They observed that the highest content of polyphenol was recovered using 60% aqueous ethanol, which was eight-fold that of the crude extract. In addition, approximately 95% of the polyphenol was present in the 60% and 80% ethanol fractions. As explained by Xi et al. [17], pure ethanol increases the desorption of some impurities, but polyphenols are not completely dissolved at lower ethanol concentrations. Leyton et al. [11] obtained similar results in a comparison of different Amberlite XAD resins for purification of phlorotannins from *Macrocystis pyrifera*, and XAD 16 N showed good results with a desorption ratio of 38.2%.

The adsorption process consists of different stages and does not remain stable during the adsorption phase. Generally, target molecules are absorbed through their mass transfer from the boundary layer, diffusion into the pores of the adsorbent, and/or adsorption at the surface-active sites of the adsorbent [18]. The adsorption rate is directly correlated with the duration, solvent, and adsorbent material used.

**Figure 1:**
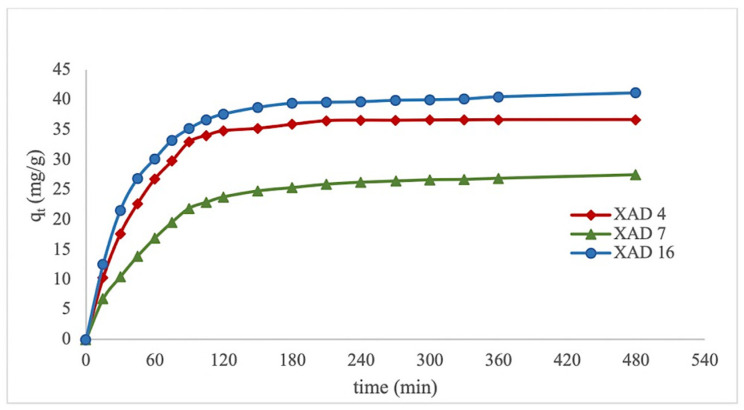
Adsorption kinetic curve of polyphenols from extracts of roasted hazelnut skin with Amberlite microporous resins.

The adsorption kinetics of polyphenols from the roasted hazelnut skin extracts obtained from the three Amberlite macroporous resins are shown in Figure 1. The adsorption quantity increased over time and adsorption was faster in the initial stages. The TPC value decreased by approximately 50% during the first 30 min by XAD 16 and XAD 4, and 1 h by XAD 7. After 1 h, the rate of adsorption gradually decreased; after 120 min of contact, only a minor change was observed because the surface binding sites of the macroporous resin were mostly saturated. The system may have reached equilibrium after 120 min. This trend is similar to that reported by Park and Lee [19]. Le et al. also observed that the adsorption equilibrium of polyphenols (sinapine) from rapeseed meal protein isolate by-products was obtained after 120 min on Amberlite XAD 16 resin [19]. Hou and Zhang reported that polyphenol adsorption equilibrium was reach after 4 h using a highly polar resin (NKA–II) [9]. The surface area of this resin is very close to that of XAD 16 and XAD 4; hence, use of nonpolar or slightly polar resin accelerates the adsorption process. As shown in Table 5, the adsorption kinetics are not best-described by a pseudo-first-order model because the absorbance capacity values were inconsistent with the values predicted using the first-order model [19].

In contrast, there was good agreement between the experimental and calculated absorbance capacities predicted by the pseudo-second-order model, and the correlation coefficients were close to unity (R^2^ > 0.99) for all types of resins. This suggests that the pseudo-second-order model can be applied to predict the kinetics of polyphenol adsorption from hazelnut skin extract using macroporous resins.

In addition, previous studies reported that the pseudo-second-order model is suitable for the adsorption of polyphenols from extracts [20,21,22]. Wang et al. reported that the adsorption patterns of polyphenols from *Eucommia ulmoides* Oliv. leaves on HPD-300, HPD-600, D-3250, X-5, D-140, NKA-9, D-101, and AB-8 resins fit well to pseudo-second-order kinetics [10]. Soto et al. also demonstrated that the pseudo-second-order model fits better than the pseudo-first-order model with the adsorption process of phenols from wine vinasses by SP700 and XAD16 HP resins. They suggested that the process fits pseudo-second-order models better when the sorption system is controlled by chemisorption mechanisms [23].

### 3.2. Dynamic Adsorption/Desorption

**Figure 2:**
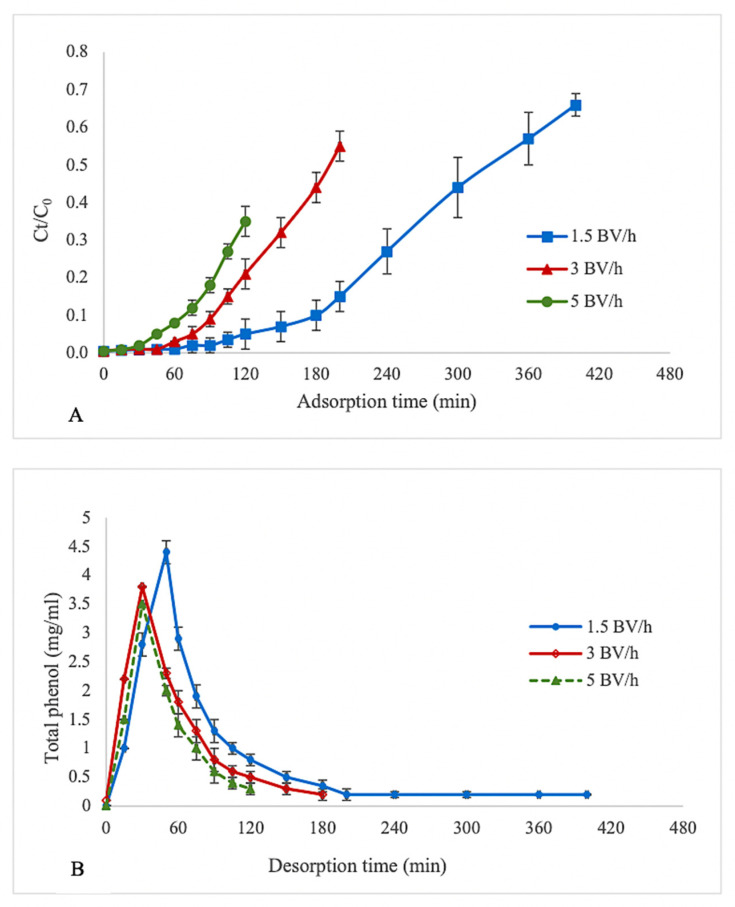
Effect of different flow rates on the (**A**) breakthrough adsorption curve and (**B**) desorption curve of polyphenols using XAD 16 resin.

The effect of the feed flow rate on the adsorption of polyphenols by XAD 16 resin is shown as a breakthrough curve in Figure 2A. The breakthrough point (BP) was obtained when the ratio of the TPC value of outlet extract (C_o_) was 5% of the TPC value of inlet extract (C_i_). Generally, BP is considered as the completion time of adsorption in industrial applications, as the adsorption capacity of the resin decreases and the absorbent cannot hold all target molecules, and thus, the solute begins to leak [24]. The best dynamic adsorption performance of the resins was obtained using the lowest flow rate (1.5 BV/h), where the BP was achieved after 120 min. At higher flow rates of 3 and 5 BV/h, the BP was reached more quickly after 75 and 45 min than at slower flow rates. These results indicate that increasing the flow rate negatively affects the dynamic adsorption of polyphenols on XAD 16 resin because as the eluent passes faster through the column, target molecules have less time to interact with active sites on the resin surface. A slow flow rate would positively impact the adsorption capacity of resins and prolong the breakthrough time [21,25,26]. Xi et al. reported the same trend for the adsorption of polyphenols from sweet potato leaves using AB-8 resin. This resin is slightly polar, with an average diameter similar to that of XAD 16. At higher flow rates, some polyphenols leaked out without being adsorbed by the resin because of the high flow speed [17]. Soto et al. showed that by increasing the flow rate from 1 to 2.5 and 5 mL/min, breakthrough decreased, and thus, the efficiency of polyphenol sorption from wine vinasses was reduced for both XAD16 HP and SP700 polymeric resins [23].

Dynamic desorption was performed after the adsorption stage of hazelnut skin extract when the BP was obtained and using ethanol solution (70% *v*/*v*) at three flow rates (1.5, 3, 5 BV/h). The desorption curves are shown in Figure 2B. Higher polyphenol recovery was observed at a desorption flow rate of 1.5 BV/h. By increasing the flow rate, the time required to recover a higher quantity of polyphenols was reduced. Similarly, Li et al. [27] and Park and Lee [21] indicated that a higher flow rate can shorten the time required to reach maximum recovery. Based on the results, using 5 g of XAD 16 resin, 87.7% of polyphenols was recovered from hazelnut skin extract with a desorption ratio of 92.36% by eluting 10 BV of ethanol solution (70% *v*/*v*) as solvent at adsorption and desorption flow rates of 1.5 BV/h. Hou and Zhang recovered 85.74% of total phenol in *Vernonia patula* extract using NKA-II resin, which is 2.48-fold higher than the total phenol of the crude extract [9]. Vavouraki showed that FPX66 resin absorbed 60% of phenolic compounds from olive mill wastewater and, using a solvent mixture of ethanol/isopropanol (1:1), recovered 70% of polyphenols [28].

### 3.3. Phenolic Content and Antioxidant Activity

A comparison between the phenolic content and antioxidant activity of the hazelnut skin extract before and after purification with the dynamic adsorption/desorption phases is shown in Table 6. Among the polyphenolic compounds identified in hazelnut skin, gallic acid and protocatechuic acid belong to subclasses of phenolic acids, (+)-catechin and (−)-epicatechin belong to the subclass of flavan-3-ols, and quercetin belongs to flavonols. These compounds were selected, identified, and quantified in both the initial and purified extracts.

The levels of all phenols were increased in the purified extract. The concentrations of gallic acid and protocatechuic acid in the purified extract increased by approximately three-fold, whereas catechin, epicatechin, and quercetin were increased by over five-fold compared to those in the crude extract. Hou and Zhang reported that the contents of chlorogenic acid and caffeic acid in *V. patula* extracts were increased 2.5-fold after column desorption from NKA-II resin using 5.5 BV of 60% ethanol at a flow rate of 3 BV/h [9]. Johnson and Mitchell showed that Amberlite resins assist in the debittering of olives during normal brine storage by adsorbing bitter phenols. They reported higher adsorption of oleuropein, ligstroside, and oleacein on FPX66 and XAD 16 N resins than on XAD 7 HP and XAD 4 resins [29]. Zheng and Wang utilized AB-8 resin to purify anthocyanins from *Aronia melanocarpa* fruits. The anthocyanin purity increased by 11.5-fold in the final product, using 80% ethanol as desorbing solvent at an elution flow rate of 2.0 BV/h [30].

The results of antioxidant activity were in accordance with the levels of phenolic compounds, with DPPH and ABTS values significantly higher after dynamic adsorption/desorption processes than before these processes, confirming that phenolic compounds were present in the purified extract.

## 4. Conclusions

The adsorption/desorption conditions of the three Amberlite resins were evaluated to optimize the extraction and purification of polyphenols from the ethanol extract of roasted hazelnut skin. Static adsorption and desorption tests showed that 5 g of Amberlite XAD16 resin had the highest adsorption capacity (40.06 ± 0.55 mg GAE/g) and adsorption ratio. The adsorption kinetics were well-fitted by a pseudo-second order model. Among the tested concentrations of desorbing solvent, 70% *v/v* ethanol solution showed the highest desorption ratio (81.17 ± 1.19%). In the dynamic adsorption/desorption processes performed using 5 g of XAD 16, the breakthrough point increased with decreasing adsorption flow rates, whereas the higher flow rate of solvent in dynamic desorption shortened the desorption time, but polyphenol recovery (87.7%) was observed at the lowest flow rate (1.5 BV/h). The purified extract showed higher phenolic compound levels and antioxidant activity than the crude extract and may be useful as a natural source of bioactive compounds for producing functional foods, as well as cosmetic and pharmaceutical preparations.

## Figures and Tables

**Table 1:** Physicochemical characteristics of Amberlite resins used for the recovery of polyphenolic compounds by a roasted hazelnut skin extract.

	XAD 4	XAD 7	XAD 16
Polarity	non polar	moderately polar	non polar
Chemical structure	Hydrophobic polyaromatic	Acrylic ester	Hydrophobic polyaromatic
Dry density (g/mL)	1.08–1.02	1.24–1.05	1.08–1.02
Surf. Area (m^2^/g)	725	450	900
Pore diameter (nm)	5	9	10
Pore mesh size	20–60	20–60	20–60
Pore volum (mL/g)	0.98	1.14	0.82
Particle size (mm)	0.3–1.2	0.3–1.2	0.3–1.2

**Table 2:** Adsorption ratios (A%; mean ± standard deviation) of polyphenols from an extract of roasted hazelnut skin by different resin types and amount and results of ANOVA with Duncan’s test.

	Resin Amount (g)					
	1	2	3	4	5	Significance
XAD 4	13.87 ± 0.74 ^Be^	24.82 ± 1.20 ^Bd^	34.82 ± 0.58 ^Bc^	51.89 ± 0.79 ^Bb^	58.73 ± 0.75 ^Ba^	***
XAD 7	10.95 ± 1.16 ^Ce^	20.65 ± 0.55 ^Cd^	24.25 ± 1.03 ^Cc^	38.23 ± 1.09 ^Cb^	42.66 ± 0.89 ^Ca^	***
XAD 16	15.58 ± 0.47 ^Ae^	29.03 ± 1.87 ^Ad^	38.93 ± 1.95 ^Ac^	58.81 ± 1.17 ^Ab^	65.06 ± 0.14 ^Aa^	***
Significance	***	***	***	***	***	

Means in each column with the same uppercase letter are not significantly different according to Duncan’s test (*p* < 0.05); means in each row with the same lowercase letter are not significantly different according to Duncan’s test (*p* < 0.05); *** *p* < 0.001.

**Table 3:** Adsorption capacity (q_a_; mean ± standard deviation) of polyphenols from an extract of roasted hazelnut skin by different resin types and amount and results of ANOVA with Duncan’s test.

	Resin Amount (g)					
	1	2	3	4	5	
XAD 4	17.70 ± 0.95 ^Be^	20.82 ± 0.36 ^Ad^	23.38 ± 0.39 ^Bc^	26.61 ± 0.40 ^Bb^	36.14 ± 0.46 ^Ba^	***
XAD 7	13.97 ± 1.48 ^Cd^	15.25 ± 0.40 ^Bd^	16.28 ± 0.69 ^Cc^	19.60 ± 0.56 ^Cb^	26.25 ± 0.54 ^Ca^	***
XAD 16	19.88 ± 0.61 ^Ae^	22.39 ± 1.44 ^Ad^	26.14 ± 1.30 ^Ac^	30.16 ± 0.60 ^Ab^	40.05 ± 0.55 ^Aa^	***
Significance	***	***	***	***	***	

Means in each column with the same uppercase letter are not significantly different according to Duncan’s test (*p* < 0.05); means in each row with the same lowercase letter are not significantly different according to Duncan’s test (*p* < 0.05); *** *p* < 0.001.

**Table 4:** Static desorption ratios (mean ± standard deviation) of XAD 16, XAD 4, and XAD 7 Amberlite resins using different ethanol solutions, and ANOVA results with Duncan’s test.

	Ethanol Concentration (%)	Resin Amount (g)					Significance
		1	2	3	4	5	
XAD 4	99.99	35.27 ± 0.48 ^Be^	44.70 ± 1.41 ^Bd^	58.46 ± 0.98 ^Bc^	65.85 ± 2.90 ^Bb^	72.53 ± 0.78 ^Ba^	***
	70	40.70 ± 2.04 ^Ad^	51.07 ± 1.94 ^Ac^	64.26 ± 1.66 ^Ab^	73.94 ± 1.45 ^Aa^	76.64 ± 0.93 ^Aa^	***
	50	32.40 ± 1.18 ^Ce^	40.28 ± 1.21 ^Cd^	50.37 ± 1.70 ^Cc^	58.17 ± 2.28 ^Cb^	65.10 ± 1.93 ^Ca^	***
Significance		***	***	***	***	***	
XAD 7	99.99	14.73 ± 0.46 ^Ce^	19.57 ± 0.37 ^Cd^	30.26 ± 0.77 ^Cc^	38.34 ± 3.13 ^Cb^	43.58 ± 1.43 ^Ca^	***
	70	17.76 ± 0.91 ^Be^	29.92 ± 0.77 ^Bd^	37.49 ± 0.79 ^Bc^	44.46 ± 0.92 ^Bb^	48.75 ± 2.13 ^Ba^	***
	50	19.89 ± 0.59 ^Ae^	33.45 ± 1.08 ^Ad^	40.57 ± 0.75 ^Ac^	48.64 ± 0.83 ^Ab^	54.37 ± 1.65 ^Aa^	***
Significance		***	***	***	***	***	
XAD 16	99.99	39.46 ± 1.50 ^Be^	47.49 ± 1.19 ^Bd^	58.97 ± 1.23 ^Bc^	71.38 ± 2.28 ^Bb^	75.80 ± 2.35 ^Ba^	***
	70	45.06 ± 1.47 ^Ae^	53.05 ± 2.31 ^Ad^	65.65 ± 1.22 ^Ab^	76.79 ± 2.41 ^Ab^	81.17 ± 1.19 ^Aa^	***
	50	35.66 ± 0.70 ^Ce^	43.89 ± 0.66 ^Cd^	53.28 ± 0.92 ^Cb^	61.99 ± 2.60 ^Cb^	67.01 ± 2.46 ^Ca^	***
Significance		***	***	***	***	***	

Means with same uppercase letter are not significantly different between ethanol concentration for each resin type, according to the Duncan’s test (*p* < 0.05); means in each row with the same lowercase letter are not significantly different according to Duncan’s test (*p* < 0.05); *** *p* < 0.001.

**Table 5:** Kinetic parameters of static adsorption phase evaluated using two model equations.

		Pseudo-First Order			Pseudo-Second Order		
	q_e_ exp.	k_1_	q_e_	r^2^	k_2_	q_e_	r^2^
XAD 4	36.68	0.0210	33.08	0.9853	0.00065	38.17	0.9947
XAD 7	27.49	0.0105	18.43	0.9560	0.00065	29.07	0.9972
XAD 16	41.15	0.0105	20.12	0.9014	0.00061	41.32	0.9950

**Table 6:** Concentration (mean ± standard deviation) of polyphenolic compounds and values (mean ± standard deviation) of antioxidant activities for the extract obtained from roasted hazelnut skin before and after dynamic absorption/desorption performed with 5 g of Amberlite XAD 16 resin.

	Raw Extract	Purified Extract	Increment% *
Gallic acid (µg/mL)	9.16 ± 0.74	30.85 ± 2.01	237
Protocatechuic acid (µg/mL)	2.80 ± 0.22	8.18± 0.12	192
Catechin (µg/mL)	4.39 ± 0.34	22.06 ± 0.44	402
Epicatechin (µg/mL)	2.72 ± 0.31	14.83 ± 1.05	445
Quercitin (µg/mL)	9.34 ± 0.76	47.23 ± 2.25	406
DPPH (mM TE/mL)	13.44 ± 0.85	83.51 ± 1.25	521
ABTS (mM TE/mL)	8.71 ± 1.23	51.83 ± 1.45	495

* (amount in purified extract-amount in raw extract) * 100/amount in raw extract.

## Data Availability

Not applicable.
